# Outcomes of Surgical Management of Turf Toe: 12-Year Results

**DOI:** 10.7759/cureus.57808

**Published:** 2024-04-08

**Authors:** Natalie Limaye, Mohit Sethi, Brijesh Ayyaswamy

**Affiliations:** 1 Medical Education, University of Liverpool, Liverpool, GBR; 2 Orthopaedics and Trauma, North Tees and Hartlepool NHS Foundation Trust, Stockton, GBR

**Keywords:** mri, manchester oxford foot and ankle score, metatarsophalangeal joint, magnetic resonance imaging, gleno-sesamoid complex, turf toe

## Abstract

Background

“Turf toe” is a classical capsuloligamentous injury to the plantar surface of the metatarsophalangeal (MTP) joint of the great toe. The name is synonymous with injuries sustained on artificial turf or hard grounds. The classical injury pattern is a hyperdorsiflexion injury with an axial load. The outcomes of these injuries are unpredictable and there are no clear guidelines for the management of these injuries. These injuries are debilitating and can lead to long-term problems and inability to return to pre-injury activity level if missed. We present a long-term surgical follow-up of severe grade 3 turf toe injuries.

Methods

In the period from 2011 to 2022, we treated 20 patients with turf toe/MTP joint instability. There were 10 football injuries (50%), six running injuries (30%), two gymnastic injuries (10%), one motorcycle injury (0.5%), and one was a ballet dancer (0.5%). All the grade 1 and 2 injuries were treated conservatively with rest, ice application, and splinting of the toe. Grade 3 injuries were treated surgically and strict rehabilitation protocol was followed.

Results

The mean age at surgery was 32.7 years and the average patient follow-up was 7.5 months after surgery. The Manchester-Oxford Foot Questionnaire (MOXFQ) score showed a statistically significant improvement from a mean of 73.0 (median = 75) preoperatively to 28.1 (median = 28.6) postoperatively (median improvement = 46.4, P = 0.022). Similarly, there was a significant improvement in pain score, which showed an improvement from a mean of 72.9 (median = 70.0) preoperatively to a mean of 22.9 (median = 25.0) postoperatively (median improvement = 51.3, P = 0.022).

Conclusion

Turf toe is a serious injury that may prevent a high percentage of patients from resuming their previous physical activities. The correct identification, classification, and grading of the first MTP joint instability helps in decision-making and achieving good surgical outcomes.

## Introduction

Turf toe injury is a hyperextension injury of the great toe at the metatarsophalangeal (MTP) joint ligament complex, including the capsule, sesamoids, plantar plate, and tendons. This then leads to attenuation or disruption of the capsular ligamentous complex supporting the joint. The first MTP joint has a complex anatomy as compared to lesser toes. It has a complex arrangement of ligaments, bone, and tendons on the plantar surface. These injuries lead to prolonged morbidity and are seldom underdiagnosed. This injury often happens to athletes involved in sports on artificial turf, hence the name "turf toe." It can also arise from a variety of activities involving any artificial hard surface. Artificial turf is harder than grass and does not flex as much as real grass when subjected to pressure. This together with the wearing of soft-soled shoes are the predisposing factors for turf toe [1]. It also happens in football when a foot is fixed to the ground with the heel elevated, and a player lands on the back of the foot, causing injury to the hallux MTP joint [2].

Turf injuries are divided into three grades as described by McCormick and Anderson [3] depending on the mechanism and extent of injury. Grade 1 is a sprain or attenuation of the plantar capsular ligamentous complex of the hallux MTP joint, grade 2 is a partial rupture of the plantar soft tissue structures of the hallux MTP joint, and grade 3 is a complete rupture of the plantar structures of the hallux MTP joint. As per the literature, grade 1 and 2 injuries are usually treated conservatively whereas there is equivocal evidence for grade 3 injuries being treated conservatively or surgically. The outcome usually depends upon the grade of injury, type of treatment, and physiotherapy regime.

Due to the nature of the injury and involved complexity, it can lead to persistent pain and loss of push-off strength. In the longer run, these injuries can lead to chronic joint pain, progressive deformity, and arthritic changes. To treat these injuries, it is imperative to take precise history and supplement it with thorough clinical and radiologic examination. Physical examination includes pain and instability on hyperextension and valgus stress. The radiologic examination includes getting weight-bearing anteroposterior, lateral, and axial sesamoid views. When compared to the contralateral side, a difference of more than 3 mm between the metatarsal head and the medial sesamoid is indicative of injury to the plantar plate sesamoid complex at the base of the proximal phalanx. When suspected, an MRI scan should be done to look at soft tissue injury.

The aim of this study was to present the 12-year results of treating 18 cases of traumatic capsuloligamentous complex injuries of the first MTP joint with an average follow-up of six years (two years to 12 years).

## Materials and methods

We retrospectively analyzed patients receiving turf toe surgery over a 12-year period, between 2011 and 2022. All the patients were assessed clinically and graded according to signs as described by McCormick and Anderson in their classification [3]. All the patients in the study underwent a standardized process of care starting with a minimum of six months of conservative treatment prior to the surgery. In the acute phase, this consisted of rest, ice, compression, and elevation (RICE). The toe was supported using a standard toe alignment splint for three months in addition or in isolation in the initial three months. After the acute phase, taping the great toe to the lesser toes to prevent movement of the MTP joint, modifications to footwear, and toe extension flexion exercises were tried.

**Figure 1:**
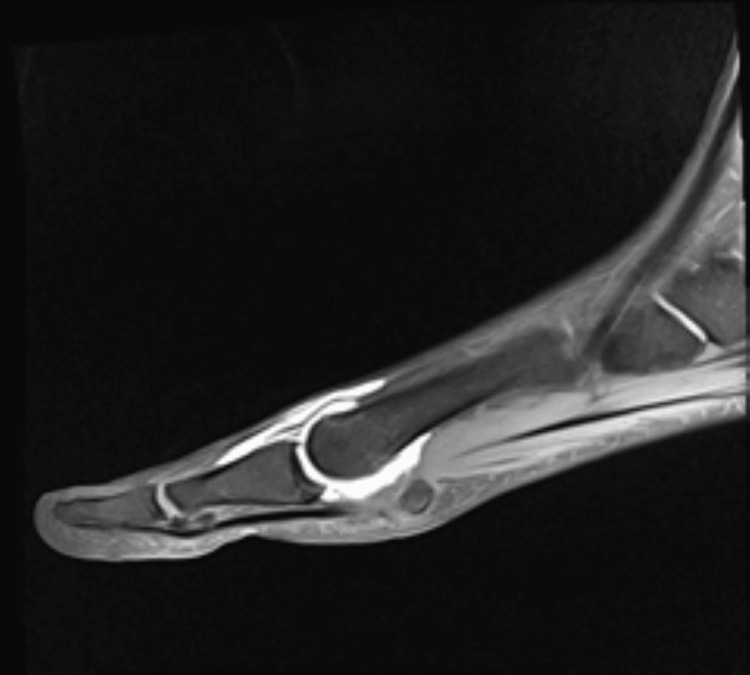
MRI scan with contrast of the great toe metatarsophalangeal joint showing rupture of the capsular ligamentous complex MRI arthrogram of a 32-year-old male patient presenting with hyperextension injury of the great toe.

**Figure 2:**
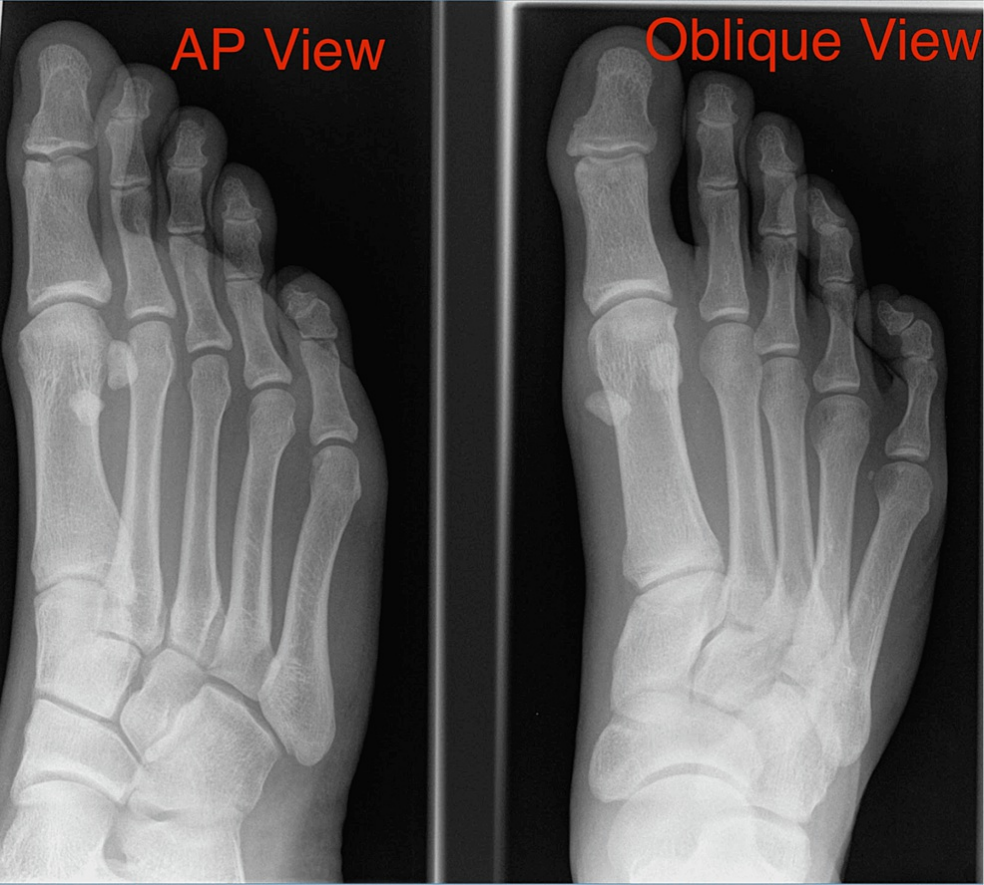
X-ray of the right foot showing subluxation of the first metatarsophalangeal joint in oblique view X-rays of a 32-year-old male patient with hyperextension injury of the great toe. Oblique X-rays showing subluxation of the first metatarsophalangeal joint suggestive of a turf toe injury.

Patients were carefully chosen by history and thorough clinical examination and diagnosis was confirmed in all patients with radiographs and magnetic resonance imaging. Figure 1 shows an MRI scan with contrast showing rupture of capsuloligamentous structures. Figure 2 shows the subluxation of the MTP joint in the oblique view. Patients with grade 1 and 2 injuries were treated conservatively whereas patients with grade 3 injuries were given the option of surgical repair. All cases were operated on by a single foot and ankle surgeon.

Inclusion criteria included patients between the ages of 18 and 75 years, patients with positive evidence of soft tissue injury on imaging, and exclusive traumatic injury. Patients with rheumatoid arthritis, diabetes, and smokers were excluded from the study.

All the patients were operated under general anesthesia and an ankle anesthetic block. All surgeries were performed under tourniquet control. Dorsoplantar incision was used and full-thickness flaps were created to avoid damaging the blood supply and avoid healing problems. Devitalized tissue was debrided and repair was performed using one suture anchor at the base of the proximal phalanx to reattach the gleno-sesamoid complex. In three patients, a dorsal cheilectomy was also done at the same time. This was due to the early presentation of hallux rigidus with the chronic turf toe. These patients were excluded from the study to avoid bias. The incision was closed in layers with Vicryl and nylon.

Postoperatively, patients were given a heel weight-bearing shoe for 14 days. After removing sutures on the 14th day, weight-bearing was allowed and a toe alignment splint was given for another four weeks. Physiotherapy was then commenced and continued for a minimum of six weeks. The aim was to achieve 30-40 degrees of painless dorsiflexion before commencing on to strenuous and high-demand sporting activities.

To assess the outcome, we used the Manchester-Oxford Foot Questionnaire (MOXFQ) [4], which is a self-administered 16-item questionnaire. Patients were assessed using the questionnaire before and after surgery and comparison was made in all of the three domains of the questionnaire, i.e., walking/standing, pain, and social interaction.

Statistics were obtained using SPSS for Windows (IBM Corp., Armonk, NY). The data were checked for normality and this confirmed a non-normal distribution. Therefore, the non-parametric Wilcoxon signed-rank test for paired data was used to test for statistical significance between the pre and postoperative scores. The median in addition to the mean was reported. Statistical significance was set at P < 0.05.

## Results

We have identified 20 patients in this study, with 12 males and eight females. The mean age at surgery was 32.7 years (median = 33 years; range = 19-52 years). The mean duration of follow-up was 7.5 months (median = 7.5, range = six to nine months). Of the 20 patients, 17 were athletes. Of the 20 patients, 18 had a history of hyperextension injury to the great toe. There were 10 football injuries (50%), six running injuries (30%), two gymnastic injuries (10%), one motorcycle injury (0.5%), and one was a ballet dancer (0.5%). The most common injury mechanism was a plantar force against an obstacle, i.e., football, ground, or wall. The injury mechanism in all patients was hyperextension force with or without valgus force. Two patients (one road traffic accident and one runner) were unsure about the injury mechanism. Table 1 shows the mechanism of injury in all patients who underwent treatment.

**Table 1:** Mechanism of injury

Football injury	10 (50%)
Running	6 (30%)
Gymnastics	2 (10%)
Road traffic accident	1 (0.5%)
Ballet dancing	1 (0.5%)

The MOXFQ walking/standing domain score showed a statistically significant improvement from a mean of 73.0 (median = 75) preoperatively to 28.1 (median = 28.6) postoperatively (median improvement = 46.4, P = 0.022). The pain domain score showed a statistically significant improvement from a mean of 72.9 (median = 70.0) preoperatively to a mean of 22.9 (median = 25.0) postoperatively (median improvement = 51.3, P = 0.022). The social interaction score showed a statistically significant improvement from a mean of 62.5 (median = 56.3) preoperatively to 15.1 (median = 18.8) postoperatively (median improvement = 47.1, P = 0.022).

The MOXFQ summary index score showed a clinically and statistically significant improvement from a mean of 70.3 (median = 67.2) preoperatively to 23.4 (median = 25.0) following the repair (median improvement = 48.3, P = 0.022). The mean time to return to normal activity after the surgery was 4.0 months (median = 3.0 months). There were no cases with any complications or recurrence of symptoms. Out of 17 athletes, 16 patients were able to go back to the previous level of activity. One patient complained of residual pain and is still under follow-up. Table 2 shows improvement in patient-reported outcome measures (PROMs) following surgery.

**Table 2:** Patient-reported outcome measures (PROMs) before and after surgery The table shows improvement in patient-reported outcome measures after surgery. MOXFQ: Manchester-Oxford Foot Questionnaire.

	Preoperative	Postoperative	P-value
MOXFQ score	73.0 (median = 75)	28.1 (median = 28.6)	0.022
Pain domain score	72.9 (median = 70)	22.9 (median = 25)	0.022
Social interaction score	62.5 (median = 56.3)	15.1 (median = 18.8)	0.022

## Discussion

The term "turf toe" was first introduced by Bowers in 1976 following the observation of 27 such injuries at the University of West Virginia, coinciding with the installation of early artificial turf. Bowers attributed the injury to the combination of rigid playing surfaces and flexible footwear [1]. Subsequent reports by Coker and colleagues at the University of Arkansas highlighted similar incidents, with turf toe resulting in more missed game time compared to ankle sprains, despite ankle injuries being more prevalent [2]. Clanton's analysis of Rice University players from 1971 to 1985 estimated approximately 4.5 cases of turf toe per season, with players missing about a week on average [3]. Rodeo's investigation into American professional football players revealed a high incidence of perceived turf toe injuries, particularly on artificial turf. These early studies contributed to understanding turf toe mechanisms and raised concerns about the risk factors associated with flexible footwear on unforgiving surfaces [3]. Recent research indicates a decline in turf toe incidence among high-level college and professional football players. At the 2006 National Football League (NFL), 11% of athletes reported turf toe injuries, with running backs, tight ends, and linebackers being the most affected [5]. A review spanning five college football seasons from 2004 to 2009 found that turf toe accounted for 83% of reported injuries, with higher rates observed in division I, during games, and on artificial turf [6]. A previous study on foot injuries in UK rugby players noted that turf toe constituted 11% of all foot injuries observed over four seasons [7].

In the literature, conservative treatment is recommended for all grade 1 and 2 injuries, and equivocal evidence for grade 3 injuries. In our study, we resorted to surgical management for all grade 3 injuries. Indications for operative management are large capsular avulsion with an unstable joint, diastasis or retraction of sesamoids, vertical instability, traumatic hallux valgus deformity, chondral injury, intra-articular loose body, sesamoid fracture, and failed conservative treatment [6-8]. There are multiple articles describing the condition, presentation, and clinical assessment as well as treatment options and surgical techniques [3,8,9], but there is not much that could be found regarding pain and functional outcomes of surgical treatment over a reasonable period of follow-up [10].

Turf toe is a sprain of the MTP joint due to a hyperextension injury of a dorsi flexed toe and a plantar-flexed foot against a ground surface with the contribution of some axial loading. The plantar-plate complex is designed to resist dorsiflexion of the first MTP joint and the degree of damage depends on the overall severity of the injury and forces to the joint. A grade I sprain represents only a microtear of the ligament but the first MTP joint remains competent and can still resist dorsiflexion. A complete dorsal dislocation typically results in the complete disruption of many of these important plantar structures, including the plantar plate [5]. The classification of injury, as described by McCormick et al., is directly proportional to the anatomical relationships of the MTP joint [3]. On clinical examination, the toe is assessed for swelling, ecchymosis, and deformity. The anteroposterior drawer test of the MTP joint is used to assess the integrity of the plantar plate and sesamoid complex [11]. Comparative anteroposterior, lateral, and axial views should be compared with the normal side to rule out subtle injuries. One should pay attention to indirect signs to rule out capsuloligamentous injuries. Waldrop et al. have suggested taking a 45-degree extension stress view to improve the sensitivity [12,13].

MRI is the gold standard and it helps identify the acute injury as well as the inflammatory process in the capsuloligamentous structures [14]. Arthroscopy has been described as the best diagnostic and therapeutic tool. It is still emerging and has a steep learning curve. In the future, with the advent of science, we will be able to treat these injuries more efficiently with all arthroscopic techniques [15,16].

In our study, we have focused on reviewing the outcome in patients undergoing surgery for turf toe using a published functional assessment tool. All patients experienced a clinically and statistically significant improvement in outcome as measured by the MOXFQ. This was not only for the summary index score but also for each of the three domains of walking/standing, pain, and social interaction. This successful outcome is substantiated by the median time for return to normal activities of four months. None of our patients suffered any complications.

The strengths of the study included a large number of patients who were assessed for a significant length of time. We present the 12-year follow-up, which has not been reported in many studies to date. There was no bias as all the patients were operated on by a single surgeon and standard surgical procedures and rehabilitation protocol were used for all patients.

This was a retrospective study and we acknowledge the fact that a larger prospective study is the need of the hour. We also did not address the outcome of surgical versus conservative management for grade 3 injuries. It is something we would like to address in our future studies.

## Conclusions

“Turf toe” is a serious injury that can lead to long-term consequences. If it is not diagnosed and treated in a timely manner, patients struggle to go back to pre-injury activity levels. The surgical outcomes of turf toe surgery have not been very successful in the past. However, we believe that accurate diagnosis and careful selection of patients are crucial for effectively managing these injuries. This study demonstrates that good patient outcomes can be obtained with early recognition and appropriate surgical treatment of turf toe injuries.

## References

[1] Bowers KD Jr, Martin RB (1976). Turf-toe: a shoe-surface related football injury. Med Sci Sports.

[2] Mullen JE, O’Malley MJ (2004). Sprains--residual instability of subtalar, Lisfranc joints, and turf toe. Clin Sports Med.

[3] McCormick JJ, Anderson RB (2010). Turf toe: anatomy, diagnosis, and treatment. Sports Health.

[4] (2016). The Manchester-Oxford Foot Questionnaire (MOxFQ). https://innovation.ox.ac.uk/outcome-measures/manchester-oxford-foot-questionnaire-moxfq.

[5] Drakos MC, Fiore R, Murphy C, DiGiovanni CW (2015). Plantar-plate disruptions: "the severe turf-toe injury." three cases in contact athletes. J Athl Train.

[6] McCormick JJ, Anderson RB (2010). Rehabilitation following turf toe injury and plantar plate repair. Clin Sports Med.

[7] George E, Harris AH, Dragoo JL, Hunt KJ (2014). Incidence and risk factors for turf toe injuries in intercollegiate football: data from the National Collegiate Athletic Association injury surveillance system. Foot Ankle Int.

[8] McCormick JJ, Anderson RB (2009). The great toe: failed turf toe, chronic turf toe, and complicated sesamoid injuries. Foot Ankle Clin.

[9] Doty JF, Coughlin MJ (2013). Turf toe repair: a technical note. Foot Ankle Spec.

[10] Anderson RB (2002). Turf toe injuries of the hallux metatarsophalangeal joint. Tech Foot Ankle Surg.

[11] Mason LW, Molloy AP (2015). Turf toe and disorders of the sesamoid complex. Clin Sports Med.

[12] Waldrop NE 3rd, Zirker CA, Wijdicks CA, Laprade RF, Clanton TO (2013). Radiographic evaluation of plantar plate injury: an in vitro biomechanical study. Foot Ankle Int.

[13] Favinger JL, Porrino JA, Richardson ML, Mulcahy H, Chew FS, Brage ME (2015). Epidemiology and imaging appearance of the normal Bi-/multipartite hallux sesamoid bone. Foot Ankle Int.

[14] Schein AJ, Skalski MR, Patel DB (2015). Turf toe and sesamoiditis: what the radiologist needs to know. Clin Imaging.

[15] Lui TH (2008). Stabilization of first metatarsophalangeal instability with plantar plate tenodesis. Foot Ankle Surg.

[16] Lui TH (2015). First metatarsophalangeal arthroscopy in patients with post-traumatic hallux valgus. Foot (Edinb).

